# Motor modules during adaptation to walking in a powered ankle exoskeleton

**DOI:** 10.1186/s12984-017-0343-x

**Published:** 2018-01-03

**Authors:** Daniel A. Jacobs, Jeffrey R. Koller, Katherine M. Steele, Daniel P. Ferris

**Affiliations:** 1 Department of Mechanical Engineering, Temple University, 1947 N. 12th Street, Philadelphia, PA USA; 2 Department of Mechanical Engineering, University of Washington, 3900 E Stevens Way NE, Seattle, WA USA; 3 Department of Mechanical Engineering, University of Michigan, 2350 Hayward St, Ann Arbor, MI USA; 4 Department of Biomedical Engineering, University of Florida, 1275 Center Drive, Gainesville, FL USA

**Keywords:** Neuromechanics, Gait, Adaptation, Motor module, Bilateral assistance, Exoskeleton

## Abstract

**Background:**

Modules of muscle recruitment can be extracted from electromyography (EMG) during motions, such as walking, running, and swimming, to identify key features of muscle coordination. These features may provide insight into gait adaptation as a result of powered assistance. The aim of this study was to investigate the changes (module size, module timing and weighting patterns) of surface EMG data during assisted and unassisted walking in an powered, myoelectric, ankle-foot orthosis (ankle exoskeleton).

**Methods:**

Eight healthy subjects wore bilateral ankle exoskeletons and walked at 1.2 m/s on a treadmill. In three training sessions, subjects walked for 40 min in two conditions: unpowered (10 min) and powered (30 min). During each session, we extracted modules of muscle recruitment via nonnegative matrix factorization (NNMF) from the surface EMG signals of ten muscles in the lower limb. We evaluated reconstruction quality for each muscle individually using *R*^2^ and normalized root mean squared error (NRMSE). We hypothesized that the number of modules needed to reconstruct muscle data would be the same between conditions and that there would be greater similarity in module timings than weightings.

**Results:**

Across subjects, we found that six modules were sufficient to reconstruct the muscle data for both conditions, suggesting that the number of modules was preserved. The similarity of module timings and weightings between conditions was greater then random chance, indicating that muscle coordination was also preserved. Motor adaptation during walking in the exoskeleton was dominated by changes in the module timings rather than module weightings. The segment number and the session number were significant fixed effects in a linear mixed-effect model for the increase in *R*^2^ with time.

**Conclusions:**

Our results show that subjects walking in a exoskeleton preserved the number of modules and the coordination of muscles within the modules across conditions. Training (motor adaptation within the session and motor skill consolidation across sessions) led to improved consistency of the muscle patterns. Subjects adapted primarily by changing the timing of their muscle patterns rather than the weightings of muscles in the modules. The results of this study give new insight into strategies for muscle recruitment during adaptation to a powered ankle exoskeleton.

## Background

Understanding how the central nervous system coordinates the muscles in the human body is vital to advance our understanding of pathological gait impairment, motor learning, and the effect of assistive devices [1]. One method for testing hypotheses of control structure is to express the muscle signals as a linear combination of a small set of representative functions, called modules, and examine changes in organization of those modules. Motor modules [2], task-based constraints [3], geometric organization of the musculoskeletal system [4], and optimal (or “good-enough”) control theories [5, 6] potentially influence muscle coordination and can constrain muscle signal data to a low-dimensional set of modules. While the underlying mechanisms that drive the organization of these modules are unknown, expressing muscle signals using concise representative functions can provide insight into coordination patterns, improve rehabilitation protocols and inform diagnostic algorithms by facilitating physiological comparisons across different patient populations. [7–9].

For patients with mobility problems, powered robotic devices (e.g. exoskeletons) can help restore normal gait patterns and provide feedback on mechanical and neurological performance [10]. A lack of adaptability and poor response to human intent currently limit the effectiveness of exoskeletons in rehabilitation research [11]. Investigating changes in the patterns of muscle coordination during adaptation to walking in a powered exoskeleton can help future devices provide more natural and effective assistance.

Research into the patterns of muscle coordination have shown that for many activities (e.g. walking [12, 13], running [14, 15], cycling [16, 17], and balancing [18, 19]) the activity of a set of *n* muscles, measured via surface electromyography (EMG) can be approximated by a set of *k* modules (*k*<*n*). Each muscle signal can be approximated by the linear combination of its module weightings and the corresponding timing patterns for each module.

Previous studies on the factorization of walking data have shown that 4-6 modules can explain the majority of variance in the EMG data [14, 19–22]. Several studies of the upper limb have also shown that representative modules can be used to describe horizontal reaching [23] and 3-D reaching with upper arm support [24]. Prosthetic control of upper-limb devices have also used modules to generate more natural, intuitive control schemes [25]. Research into the motor modules used during isometric force generation have shown that motor module structure is preserved during visuomotor rotation [26], and that control algorithms that are compatible with the subject’s original motor modules lead to faster adaptation to new tasks [27].

The structural properties of the extracted motor modules can also highlight differences in motor control between groups. For example, compared to a healthy population, sub-acute stroke survivors had similar timing patterns of muscle recruitment but significantly different weighting patterns [28]. A separate study on stroke survivors showed that significantly fewer modules were needed to reconstruct the muscle signals in the paretic leg compared to the non-paretic leg and healthy controls [29].

While these studies have evaluated the properties of muscle patterns during steady-state and perturbed learned movements, there is little research on the muscle patterns during adaptation to walking in an exoskeleton. Previous studies on both healthy and patient populations in exoskeletons have shown that providing assistance can both increase and decrease the muscle activity in specific muscles surrounding a single joint as well as change the recruitment patterns of muscles at other joints [30, 31]. It is not known whether these changes reflect the creation, destruction, or merging of different representative muscle recruitment patterns during adaptation to walking with powered assistance. Recently, Steele et al. (2017) investigated motor synergies under different power and torque inputs to an ankle exoskeleton [32] and found that subjects changed the timings or weightings depending upon whether the exoskeleton control algorithm was varying exoskeleton torque or work. However, it is still unclear whether these changes persist over longer durations of time and under alternative control methods. In a previous study on ankle exoskeletons, subjects saw greater reductions in the soleus and gastrocnemius and less perturbation of normal ankle kinematics when walking under myoelectric control compared to kinematic control [33]. Because myoelectric control uses muscle activity to control the exoskeleton, we hypothesized that we may see a larger influence on the motor modules than that other controllers.

The aims of this study were: 1) determine if there is a change in module organization (i.e. number of modules and module function) between powered and unpowered exoskeleton conditions, and 2) determine if the changes in the muscle recruitment with bilateral assistance are best explained by changes in timing patterns or in muscle weightings. We hypothesized that the number of modules needed to reconstruct muscle data would be the same between the unpowered and powered conditions, because the exoskeleton would perturb but not change the overall biomechanical task, similar to other studies of perturbed walking [34]. We also hypothesized that across conditions there would be greater similarity in muscle timings than weightings, because previous research on powered ankle exoskeletons has shown that the devices primarily influence the peak value and have a lesser effect on muscle recruitment shape [31].

The authors previously published the results of the kinematic, metabolic, and joint moment analyses results [35]. The published results demonstrated how the adaptation strategy can be affected by the exoskeleton control strategy. In previous studies in proportional myoelectric control in the same lab [36, 37], no differences were found in the total ankle joint moment between the powered and unpowered conditions. These prior studies also found that the changes in muscle signal occurred primarily in muscles around the ankle (soleus and tibialis anterior) but there was no change in the quadriceps or hamstring groups [36, 37]. In contrast, we found that subjects using the adaptive gain proportional myoelectric controller increased their total ankle joint moment and reduced the hip joint moment while achieving a substantial metabolic decrease. In the new controller, subjects decreased the muscle activity in both muscles spanning the ankle, hip and knee (soleus, the rectus femoris, and the biceps femoris long head).

## Methods

### Subjects

Eight healthy male subjects participated in this study: (mean + standard error of the mean) age 21 ± 1 years, height 1.80 ±.03 m, mass 74.0 ± 2.7 kg. Subjects exhibited no gait abnormalities and had no previous experience walking in a powered exoskeleton.

### Protocol

Each subject wore a custom, bilateral set of plantarflexion-assisting ankle exoskeletons, similar to previous studies [36, 37], that were created in coordination between the Human Neuromechanics Laboratory and the University of Michigan Orthotics and Prosthetics Center. All of the modifications were done by licensed technicians at the Orthotics and Prosthetic Center. The orthotics were a modified two-piece plantarflexion and dorsiflexion ankle-foot orthosis consisting of hard plastic sections connected by a stainless steel single-axis ankle joint. The lower end of the ankle joint was embedded between the midsole and the outsole of the shoe. The upper plastic cuff was lightly padded and secured around the subject’s shin by ratcheting clamps. Posterior flanges were welded to both pieces to attach the actuator. We actuated the exoskeleton using custom artificial pneumatic muscles connected to proportional pressure regulator values (MAC Values, Wixom, MI)[38].

The control algorithm for the exoskeleton was a proportional myoelectric controller with a time-varying gain [35]. Myoeletric control gives the subject direct, neurological control of the device by using the linear envelope of the subject’s electromyographic (EMG) signals as the input control signal [36, 37]. For this study, we used the subject’s left and right soleus muscles.

In classic, proportional, myoelectric control, the gain between the linear envelope of the muscle signal and the control signal is fixed. For the time-varying controller, the gain between the muscle signal and the control signal varied based on the peak soleus signal from previous 50 strides. The gain was calculated such that the average peak activation of the last 50 strides commanded the maximum actuator output. Therefore, if subjects choose to decrease their soleus signal over time due to walking with the ankle exoskeleton, they continue to receive the maximum output of the actuator due to an increase in the gain. However, in a fixed-gain controller, if the gain was selected based only on the initial soleus signal, reducing the soleus activation by 50% using the exoskeleton assistance would result in a 50% reduction in actuator command as well.

**Fig. 1:**
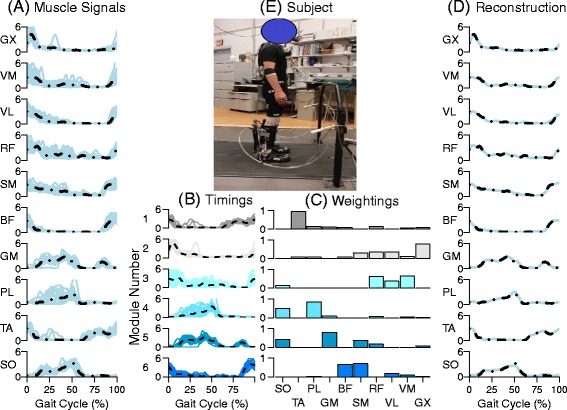
Sample module factorization of a representative subject. **a** Individual strides (light blue solid) and mean muscle signal (black dot-dash) for the ten muscle signals recorded during walking in an exoskeleton **b** Individual strides (solid) and mean value (dash) of the timing signal for the six modules extracted using nonnegative muscle factorization. **c** Weighting signals for the six modules. **d** The mean measured (light blue solid) and mean reconstructed value (black dot-dash) for the ten muscle signals. **e** Photo of the subject standing in the exoskeleton. Muscle Abbreviations: Soleus (SO), Tibialis anterior (TA), Peroneus longus (PL), Medial gastrocnemius (MG), Biceps femoris long head (BF), Semitendenosis (SM), Rectus femoris (RF), Vastus lateralus(VL), Vastus medialis(VM), and Gluteus maximus(GX)

**Fig. 2:**
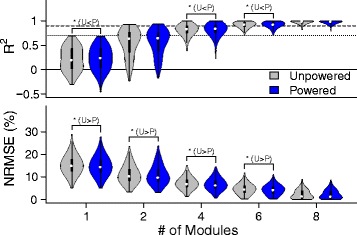
Module size selection. *R*^2^ and normalized root mean squared error (NRMSE) for module sizes of 1, 2, 4, 6, 8 for the unpowered (gray) and powered (blue) conditions. We selected six modules because the median was greater than 90% (dashed line) and the minimum was greater than 70% (dotted line). Sign tests (p < 0.05) with a significant difference in median are bracketed with a label indicating the direction of the shift. The median shifts were small relative to the median value of the group and not consistent across all sizes suggesting little difference in module organization due to assistance level. Violin plots are similar to the box and stem plots. The violin body is the probability density function which shows the estimated frequency of each point in the data set, the white dot is the median, and the black bar represents the middle two quartiles

Subjectsn[-.8cm]Figure: Journal’s standard requires that the first figure referenced in the manuscript text should be Figure 1, the second, Figure 2, etc. However, the original sequence of figure citations “1–3, 5, 4, 6–8” is out of order. Figures and its corresponding citations were reordered so that they are cited in consecutive numerical order. Please check if action taken is appropriate. Otherwise, kindly advise us on how to proceed. walked on a split belt instrumented treadmill (Bertec Corporation, Columbus, OH) at 1.2 m/s (Fig. 1). The testing protocol was split into three identical training sessions over the course of 1-2 weeks with at least one full day of rest (mean: 3.43 days, std. dev. 1.75 days) [36, 39] for consolidation. Each training session consisted of 50 min of level ground walking split between the two conditions: powered and unpowered (wearing the exoskeleton with the actuation turned off). The order of conditions was not randomized. Each subject walked for 10 min in the unpowered condition, followed by 30 min in the powered condition, then 10 min again in the unpowered condition. We analyzed the first 40 min for our modular control investigation. During each session, we collected 1 min segments of gait data every other minute for post-processing, resulting in a a total of 5 unpowered trials and 15 powered trials each session. The EMG system, the exoskeleton control system, and the motion capture system were synchronized by co-recording a square wave from a signal generator and aligning the streams in post-processing. Figure 1 shows a side view of a representative subject in the experimental setup and representative trial with the muscle signal data segmented into strides.

We partitioned each segment into strides based on the instant the vertical force exceeded 5% of body weight. We removed errors (e.g. two feet on the same force plate) by detecting if the stride time or the peak force was 1.5 times the standard deviation away from the mean of the segmented strides. Each stride was time normalized to 400 time points and then the 40 clean steps were concatenated.

### Electromyography

We used bipolar surface electrodes with an inter-electrode distance of 20 mm and an electrode diameter of 10 mm to record surface electromyography data from the subjects (SX230, Biometrics, Ltd, Newport, UK). Muscle signal data were multiplied by a gain of 1000 through an amplifier with a bandwidth of 20-460 Hz. We prepared the skin and placed our electrodes following the recommendations of the SENIAM group [40].

EMG data was collected at 1 kHz. We calculated the linear envelope of the EMG post-trial in three stages: 1) band pass filtering with a 20-450 Hz band [12, 20], 2) full-wave rectification, and 3) low-pass filtering at 10 Hz. All filters were second-order Butterworth filters with zero lag.

Originally, we recorded 16 channels of muscle signal data during walking from the right side leg and torso. Due to the relatively low magnitude of the signals from the hip muscles (tensor fasciae latae and adductor magnus) and the torso (rectus abdominus, erector spinae, and external obliques) during the walking task, we removed them from the analysis and considered the following 10 muscles: soleus (SOL), tibialis anterior (TA), peroneus longus (PER), medial gastrocnemius (MG), biceps femoris long head (BF), semitendenosis (SM), rectus femoris (RF), vastus lateralis (VL), vastus medialis (VM), and gluteus maximus (GX).

### Nonnegative matrix factorization

Researchers in the field of motor control have proposed several different methods for extracting motor modules from a set of muscle signals. Principal component analysis [20, 41, 42], independent component analysis [20], factor analysis [43], and nonnegative matrix factorization [44] have been used to calculate motor modules. We employed nonnegative matrix factorization (NNMF) because it constrains the timing signals to be nonnegative which follows the established activation dynamics of muscle.

Given a *m*×*n* matrix of EMG data, *F*, and a module set size, *k*, the NNMF algorithm calculates the reduced order factorization *F*=*TW*, where m is the number of samples, n is the number of muscle signals, and k the module size. In this analysis, each column of *F* is a time series of surface EMG data from a single muscle, *T* is a *m*×*k* matrix where each column is a representative timing pattern, and *W* is a *k*×*n* matrix of weightings where each column corresponds to the contribution of each muscle activation pattern to the final muscle signal [43–45].

We employed the NNMF routines in Matlab Ⓡ(Mathworks, Inc. Natick, MA) using an alternative least squares method [46]. Our input data for the NNMF algorithm was the first 40 clean strides from a trial during one of the sessions 1. The convergence criteria was set for relative function tolerance of 1E-12 and a step size change of 1E-12 [47]. Each factorization was replicated 12 times from randomized starting values and the best replication was chosen at the end.

Before NNMF, we normalized each muscle signal to unit variance [19]. After NNMF, we sorted each module, in ascending order, by the index of the peak timing signal.

#### Reconstruction quality measures

The selection of a reconstruction quality measure is non-trivial [13]. Variance accounted for, called VAF, is as common reported quality but can be challenging to interpret. Previous researchers have defined VAF as 100∗uncentered Pearon’s correlation coefficient [48, 49] and 100∗uncentered coefficient of determination [19, 34]. Other common measures are the centered coefficient of determination (*R*^2^) [20, 45, 50] and the normalized root mean square error (NRMSE) [51]).

Because the objective function of NNMF is to minimize the error between the measured muscle signals and the reduced-order, factorized representation, we chose the centered coefficient of determination and the normalized root mean square error as measures of reconstruction quality because they are directly related to the objective function and the error. In order to minimize confusion in the terminology, we will refer to the reconstruction quality measures directly and avoid using the term VAF.

Although the coefficient of determination has been used previously, the interpretation of the results can be challenging because selected method of calculating *R*^2^ can lead to different results in certain circumstances. For linear fits to linear data with an intercept, the calculation of *R*^2^ is consistent. However, in this case we are regressing nonlinear data where the total sum of squares is not equal to the sum of the residual sum of squares and the regression sum of squares [52].

Given a set of measured EMG data *f*_*i*_ to *f*_*n*_, and a set of predicted EMG data *x*_*i*_ to *x*_*n*_, the centered coefficient of determination and the normalized root mean square error of the NNMF reconstruction of a single muscle can be calculated as:

1 $$ R^{2} = 1 - \frac{SS_{error}}{SS_{total}} = 1 - \frac{\sum\limits_{i=1}^{n} \left(f_{i} - x_{i}\right)^{2}} {\sum\limits_{i=1}^{n} \left(f_{i} - \overline{f}\right)^{2}}  $$

2 $$ \text{NRMSE} = \frac{RMSE}{range(f)} =\frac{\sqrt{\frac{1}{n}\sum\limits_{i=1}^{n} \left(f_{i} - x_{i}\right)^{2}}}{max(f) - min(f)}  $$

When calculating *R*^2^ using Eq. 1, the range of values for *R*^2^ is (−*∞*,1] because there is no bound on the error in the numerator. A *R*^2^ value of 1 corresponds to a perfect fit and a *R*^2^ value of 0 indicates a fit equivalent to using the mean of the measured data as the estimator (i.e $x_{i} = \overline {f}$). Negative values indicate a fit worse than estimated by the mean of the measured data.

To determine if the function of the modules were similar between conditions, we calculated the similarity between the timing and weighting data of the unpowered and powered conditions using the normalized scalar product (also referred to as the Uncentered Pearson Correlation Coefficient and cosine similarity) [8, 17].

All three reconstruction quality measures, *R*^2^, NRMSE, and similarity were calculated individually for each muscle. Evaluating the muscles in a group can lead to underestimation of the number of modules needed to reconstruct muscle activity because the poor reconstructed muscles can be hidden by the group performance [13].

#### Normalization of reconstruction quality measures to random chance

For high dimensional signals, such as EMG data in synergy analyses, random similarity between the timings and weightings data is substantial. To compare the timing and weighting data across conditions, it is important to normalize the similarity, *r*^2^, and NRMSE values by the values found using a randomly generated dataset. Previous researchers have used three different techniques to generate random data: random shuffling of the experimental muscle signals [50, 53], random sampling from the muscle signal’s distribution or a chosen distribution [23, 54], and random generation of the timings and weightings [21, 43, 54, 55].

We chose the random generation scheme previous research [21, 43, 55] for our baseline because it does not assume any a priori knowledge of the amplitude distribution or the frequency content of the muscle signals. Using a uniform random generator, we generated 100 timing and weighting matrices. For each timing matrix in the random set, we estimated the optimal weighting matrix that minimized the prediction error ∥*F*−*TW*∥. We used a non-negative least-squares algorithm, identical to an iterative step of a single pass of NNMF, to estimate the optimal weighting. We calculated the *R*^2^ and NRMSE values for combination of the random timing matrix and optimized weighting matrix. Similarly, for each weighting matrix in the random set, we estimated the optimal timing matrix that minimized the prediction error.

Nonnegative matrix factorization generates the timing matrix, *T*, and the weighting matrix, *W*, that best satisfies *F*=*TW* given muscle signal data *F*, and the module size *k* using an alternating least squares approach where either the timing or the weighting matrix is fixed during the optimization. For the cases where we wanted to fix either the timing matrix, *T*, or the weighting matrix, *W*, a priori, we used a nonnegative least-squares optimization routine to generate the matching matrix that minimized the objective. For each trial, we also calculated mean similarity between the randomly generated timing and weighting matrices and the timing and weightings extracted by using NNMF on that trial.

For a given set of timings, we estimated the least squares weighting matrix, *W* to the original matrix equation *F*=*TW* and for a fixed set of weightings we estimated the least squares timing matrix of the transposed matrix equation *F*^⊤^=*W*^⊤^*T*^⊤^. We used a linear least squares algorithm with nonnegativity constraints with termination settings of 1E-12 for both function tolerance and step size criteria.

We grouped the *R*^2^, NRMSE, and similarity values of the 100 sets of randomly generated timing data and weighting data using the entire set of collected data (all subjects, sessions, trials and muscles). The Sign Test, which we used for statistical comparisons, tests for a shift in the median value between the two groups. As a result, we used the median value of the *R*^2^, NRMSE and similarity values calculated from the random data set to normalize the values in the experimental data.

We define the normalized measures, scaled $R^{2}(\overline {R^{2}})$ and scaled NRMSE $(\overline {NRMSE})$ as

3 $$\begin{array}{*{20}l} \text{Scaled} R^{2}(\overline{R^{2}}) &= 100\frac{R^{2} - c_{r}}{c_{max} - c_{r}} \end{array} $$

4 $$\begin{array}{*{20}l} \text{Scaled} NRMSE (\overline{NRMSE}) &= 100\frac{\text{NRMSE} - c_{r}}{c_{max} - c_{r}} \end{array} $$

where *c*_*r*_ denotes the median value calculated from the randomly generated data set and *c*_*max*_ denotes the maximum theoretical signal (1 for *R*^2^ and 0 for NRMSE). A scaled measure of 0 corresponds to the same value as the randomly generated data set and 1 is the maximum potential measure. The value of *c*_*r*_ was calculated for the timing and weightings separately. To scale the values extracted via NNMF, the value of *c*_*r*_ was set to zero and values outside of (0,100) were truncated to the boundaries.

### Module organization analysis

To calculate how many modules were needed for sufficient reconstruction, we ran nonnegative matrix factorization (NNMF) on each trial for a series of modules sizes from 1 to 8. Similar to previous research [20, 34, 56], we set a threshold value of *R*^2^ for sufficient reconstruction. The value of *R*^2^ is affected not just by the muscle coordination but also the number and choice of which muscles to use [55] and the use of concatenated vs averaged muscle signal data [21]. The threshold value of *R*^2^ is also different based on whether not the quality measures are calculated as a group for all the muscles in the trial or for each muscle individually [13]. We are using concatenated data which often requires more modules for a given threshold [21]. We also are using a more stringent individual-muscle criteria vs a grouped-muscle criteria because grouped muscle criteria can result in very poor reconstruction of specific muscles [13].

We split all of the subjects, trials, sessions, and muscle data into two subgroups for the powered and unpowered conditions. We calculated the minimum number of modules necessary to reconstruct the data set by evaluating the *R*^2^ value for all the data at each condition and module size (N[dataset size] =1200: 8 subjects, 3 sessions, 5 trials, 10 muscles). Previous researchers have used a range of values, such as 0.8 [8, 13], and 0.9 [57] as thresholds. We considered the minimum number of modules to be that where the median *R*^2^ value of the set selected for module size and conditions was greater than 0.9. A sample module factorization of a representative subject is shown in Fig. 1.

For each module size, we partitioned the resulting reconstruction quality measures into two subgroups for the powered and unpowered conditions. We tested each subgroup for normality using the Kolmogorov-Smirnov Test. The data sets were significantly different from normal (*p*< 0.05) and were skewed. For each module size, we used the Sign test to evaluate median differences in the *R*^2^ and NRMSE measures between the powered and unpowered conditions (*N*=1200: 8 subjects, 3 sessions, 5 trials, 10 muscles).

### Module components analysis

To determine if the motor modules were preserved at different assistant levels, we calculated the similarity between the module timings and weightings of the unpowered and powered conditions. For balance, we grouped the five trials of the unpowered condition with the last five trials of the powered condition, creating a 10x10 grid of tests within and between conditions. We removed the diagonal from the grid as they represented testing the timings and weightings from the same trial. In total we found 2160 similarity values. (*N*=2160: 8 subjects, 3 sessions, 90 tests in the grid).

Although similarity can describe the relationship between timings and weightings, it does not describe the error as directly as *R*^2^ and the normalized root mean squared error (NRMSE). In order to directly quantify how much mutual information is shared between the timings and the weightings of the powered and unpowered conditions, we calculated the *R*^2^ and NRMSE measures for three different combinations of the timing and weighting data for three groups with different assumptions as to how module timings and weightings might be shared between different trials. For each test, we formed a grid from the last 5 trials of the unpowered and powered conditions to create set of tests for each group.

#### Swapped group

In the swapped group, we tested whether timings and weightings extracted from an individual trial using NNMF could be used to reconstruct data from another trial. We define the swapped group:

5 $$\begin{array}{@{}rcl@{}} \text{Timings:} \left\{ F^{u}_{i} = T^{p}_{j}W^{u}_{i} \cup F^{p}_{j} = T^{u}_{i}W^{p}_{j} \right\} \end{array} $$

6 $$\begin{array}{@{}rcl@{}} \text{Weightings:} \left\{ F^{u}_{i} = T^{u}_{i}W^{p}_{j} \cup F^{p}_{j} = T^{p}_{j}W^{u}_{i} \right\}, \end{array} $$

where the superscripts *u* and *p* refer to the unpowered and powered conditions respectively and *i* and *j* refer to the *ith* trial of the unpowered condition in the 5×5 test grid and the *jth* trial of the powered condition 5×5 test grid respectively.

The swapped group used the data from all subjects and sessions with the unpowered and powered trials set into a test grid of size 25 (5 unpowered and 5 powered trials). The final data set was size 1200: 8 subjects, 3 sessions, 25 tests, 2 swaps(1 timing and 1 weighting).

#### Paired group

In the paired group, we tested whether a pair of trials, one of each condition, could be reconstructed by NNMF modules if they were paired such that they have the same timing matrix or the same weighting matrix.

7 $$\begin{array}{@{}rcl@{}} \text{Timings:} \left[F^{u}_{i}\quad F^{p}_{j}\right]= T \left[W^{u}_{i}\quad W^{p}_{j}\right] \end{array} $$

8 $$\begin{array}{@{}rcl@{}} \text{Weightings:} \left[\begin{array}{c} F^{u}_{i}\\ F^{p}_{j} \end{array}\right] = \left[\begin{array}{c} T^{u}_{i}\\ T^{p}_{j} \end{array}\right] W, \end{array} $$

where the superscripts *u* and *p* refer to the unpowered and powered conditions respectively and *i* and *j* refer to the *ith* timing block of the unpowered condition and the *jth* timing block of the powered condition respectively.

The paired group used the data from all subjects and sessions to calculate *R*^2^ and NRMSE along a test grid of size 100 (5 unpowered and 5 powered trials). The 2 off-diagonal 5x5 sub-grids representing the between condition tests were used. The grid values were not symmetric because of local minimums in the NNMF algorithm so we pooled the entire grid. The tests were repeated (*N*=2400: 8 subjects, 3 sessions, 100 tests) using the average *R*^2^ and NRMSE of the muscles for that trial.

#### Shared group

In the shared group, we tested whether a single shared timing or weighting matrix could reconstruct the entire data set (all 5 unpowered trials and all 15 powered trials).

9 $$\begin{array}{@{}rcl@{}} \text{Timings:} \left[\begin{array}{cccccccc} F^{u}_{1}& F^{u}_{2}&\dots & F^{u}_{5}& F^{p}_{1}& F^{p}_{2}&\dots & F^{p}_{15} \end{array}\right] = \\ T \left[\begin{array}{cccccccc} W^{u}_{1}& W^{u}_{2}&\dots & W^{u}_{5}& W^{p}_{1}& W^{p}_{2}&\dots & W^{p}_{15} \end{array}\right] \end{array} $$

10 $$\begin{array}{@{}rcl@{}} \text{Weightings:} \left[\begin{array}{c} F^{u}_{1}\\ F^{u}_{2}\\ \vdots \\ F^{u}_{5}\\ F^{p}_{1}\\ F^{p}_{2}\\ \vdots \\ F^{p}_{15}\\ \end{array}\right] = \left[\begin{array}{c} T^{u}_{1}\\ T^{u}_{2}\\ \vdots \\ T^{u}_{5}\\ T^{p}_{1}\\ T^{p}_{2}\\ \vdots \\ T^{p}_{15}\\ \end{array}\right] W, \end{array} $$

where the superscripts *u* and *p* refer to the unpowered and powered conditions respectively.

The shared group used the data from all subjects and sessions and tested the shared timings and weightings on the last 5 unpowered trials and the last 5 powered trials (*N*=2400: 8 subjects, 3 sessions, 5 unpowered and 5 powered trials, and 10 muscles)

### Statistics

All statistical analyses were performed in IBM SPSS Statistics 22b®; (IBM Corp. Armonk, NY). All tests were set at a significance level of 0.05.

We pre-tested the *R*^2^ and NRMSE results for normality and skew in order to choose the appropriate statistical test. We used Kolmogorov-Smirnov tests for normality and we characterized distributions as symmetric when the ratio of skewness to the standard error of skewness was <|1.96| [58, 59]. Our testing showed that all of the data was skewed so we used the non-parametric Sign Test to test for differences in *R*^2^ and NRMSE between the powered and unpowered conditions.

We used a linear, mixed-effects model to test the effect of training time on the *R*^2^ value during the powered condition. The trial number and the session were fixed effects and the subject was a random effect. We assumed a variance components structure and used restricted estimation maximum likelihood in the model. The input data was all of the subjects, sessions and trials of the powered condition with the average *R*^2^ of the 10 muscles in each trial (*N*=360: 8 subjects, 3 sessions, 15 trials). We set the module size to six based on the previous result for the minimum module size for adequate reconstruction and the significance level to 5%. If the fixed effects are significant, it would indicate that the time spent walking in the exoskeleton, as measured in session number and segment number within session, can explain the changes in the consistency of muscle signals.

## Results

### Module organization

The number of modules was preserved across powered and unpowered conditions. Based on our selection criteria, 6 modules were sufficient to reconstruct both the unpowered and powered condition data (Fig. 2). At this module size, a few values were beneath the *R*^2^ threshold of 0.7. However, values less than 0.7 were rare so we considered six factors satisfactory. For module sizes 4 and 5, the distribution of *R*^2^ had longer and thicker tails which meant that several individual muscles were not being reconstructed well at smaller sizes.

**Fig. 3:**
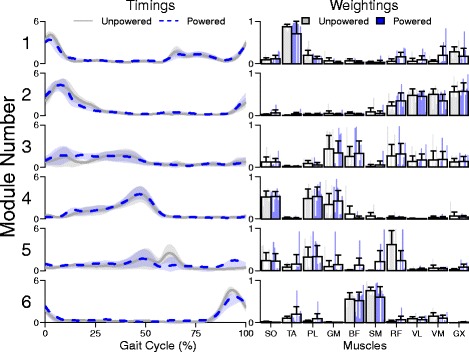
Mean subject module timing and weighting data. Normalized across subject mean and 95% confidence intervals for the timing and weighting components of the muscle recruitment modules extracted from the unpowered (gray) and powered (blue) condition data via NNMF. Individual subject weightings are shown as thin bars with the group mean and standard error in the transparent wide bar. There is strong agreement across conditions in both the timing and weighting data indicating that module organization was preserved during the adaptation to walking in an exoskeleton. Muscle Abbreviations: Soleus (SO), Tibialis anterior (TA), Peroneus longus (PL), Medial gastrocnemius (MG), Biceps femoris long head (BF), Semitendenosis (SM), Rectus femoris (RF), Vastus lateralus(VL), vastus medialis(VM), and Gluteus maximus(GX)

The module timings and weightings were also preserved across powerd and unpowered conditions. The subject-mean, step-averaged, timings and weightings of the modules extracted from the muscle data both had strong agreement between the powered and unpowered conditions. (Fig. 3).

For a module size of 6, a Sign test showed that the muscle signal data during the powered condition had slightly better median reconstruction quality than the unpowered condition for both *R*^2^ (*N*=1200, *Z*=−1.992, *p*=0.046, Powered > Unpowered, *δ*=0.002) and NRMSE (*N*=1200, *Z*=−9.324, *p*< 0.000, Powered < Unpowered, *δ*=−0.286). Similarly, at the other modules sizes, the median difference between the conditions was statistically significant but of negligible magnitude (Fig. 2).

The reconstruction quality of the extracted modules increased with training time. The linear, mixed-effects model generated from the data with a module size of six, had a significant fixed effect for session (*N*=360, *t*=7.962, *p*< 0.001, estimated value = 0.0059, 95% Confidence Interval =0.0044−0.0073) and trial (*N*=360, *t*=5.188, *p*< 0.001, estimated value =0.0007, 95% Confidence Interval =0.0004−0.0010) and the subject was not significant (*p*=.064).

**Fig. 4:**
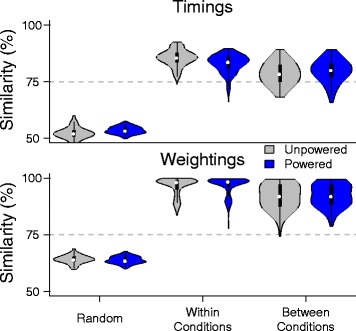
Reconstruction quality of timing and weightings across conditions compared to randomly generated baseline. *R*^2^ and normalized root mean squared error (NRMSE) of randomly generated weightings and timings for the unpowered (gray) and powered conditions (blue). The randomly generated weightings and timings could not reconstruct the muscle signals (*R*^2^≤ 0). However, the randomly generated weightings could produce a poor reconstruction (*R*^2^> 0) demonstrating that the variability in timing is harder to reproduce than in weighting

Random weightings had better reconstruction than random timings. Non-negative, least squares optimization of the estimation residual using fixed, randomly generated timing and weighting matrices led to reduced reconstruction quality compared to matrices calculated via NNMF (Fig. 4). The set of random weightings and optimized timings had a median *R*^2^ of.40 and the set of random timings and optimized weightings had a median *R*^2^ of -.048.

**Fig. 5:**
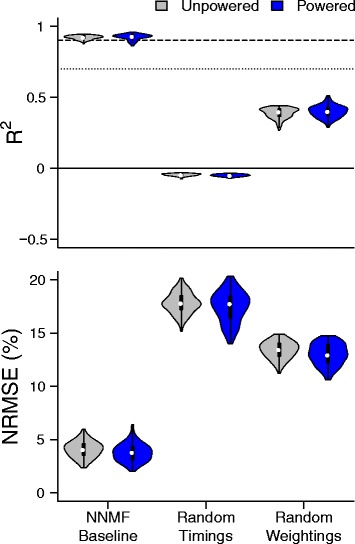
Similarity of timing and weightings across conditions compared to randomly generated baseline. Cossine Similarity of the timing and weighting matrices for the unpowered (gray) and powered conditions (blue) for three groups: randomly generated data, within condition data, and between conditions data. The threshold of similarity was set at 75% (dashed gray line). The timing and weightings were more similar than the random data set both within and between conditions. Essentially, all of the weighting data collected had similarity greater than the threshold of 75%. For the timing data, within and between conditions, the percentage of trials greater than the threshold ranged from 75.2 to 99.8% respectively

The weightings had higher similarity than the timings. The timing and weighting data was more similar both within and between conditions (unpowered, powered) than the random data (Fig. 5). For both conditions, the similarity within conditions was greater than the similarity between conditions. The similarity of the weighting data was greater than the similarity of the timing data for both conditions. A Sign test showed a large significant difference in the medians between the timings and the weightings for the concatenated data set (*N*=2160, *Z*=− 46.0240, *p*< 0.001, Weightings > Timings, *δ*=14.10).

### Module components

Evaluating the timings and weightings extracted by NNMF showed that across-group and mixed group comparisons (swapped, paired, and shared set), the weightings could explain more of the variance of the EMG data than the timings.

**Fig. 6:**
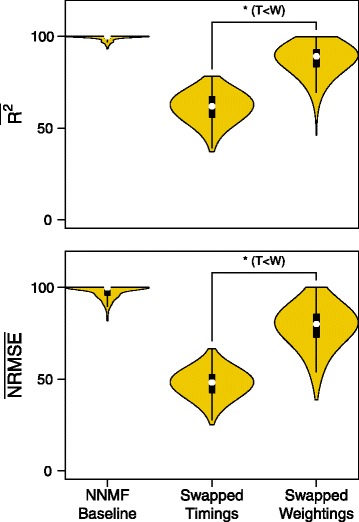
Reconstruction quality of timing and weightings swapped across conditions. Variance accounted for and Error accounted for the swapped data set, where either the timing or the matrix data of one condition was swapped with data from the opposing condition and the value normalized against random chance. A Sign test showed that the swapped weightings had significantly higher reconstruction values than the timings demonstrating that the subject’s preferred weighting varied less than timing during the adaptation period

Swapping the timing and weighting data between conditions tested if timing and weighting information was shared between conditions. The swapped weighting set had a statistically significant increase in the median variance accounted for (Sign Test: *N*=1200, *Z*=−34.497, *p*< 0.001, weightings > timings, *δ*=27.7*%*) and the error accounted for (Sign Test: *N*=1200, *Z*=−34.612, *p*< 0.001, weightings > timings, *δ*=33.2*%*) compared to the swapped timing set (Fig. 6).

**Fig. 7:**
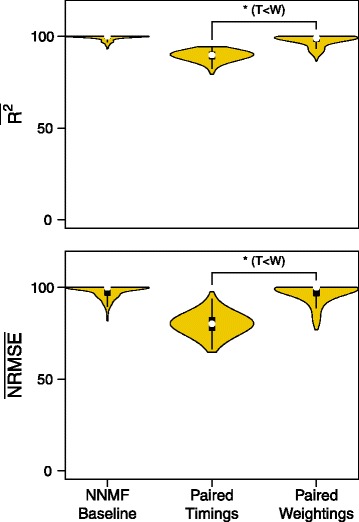
Reconstruction quality of timing and weightings paired across conditions. Variance accounted for and Error accounted for the paired data set, where either the timing or the matrix data of one condition was paired with data from the opposing condition before NNMF was applied. The resulting reconstruction quality measures were normalized against random chance. A Wilcoxon signed-rank test showed that the paired weightings had significantly higher reconstruction values than the timings demonstrating that assuming fixed weightings between conditions can produce high quality reconstructions

The paired weighting set tested whether a single representative set of timing or weighting data could explain the variance of a mixed data set consisting of EMG signals from both the powered and unpowered conditions. The paired weighting set had a statistically significant increase in the median variance accounted for (Sign Test: *N*=1200, *Z*=−34.612, *p*< 0.001, weightings > timings, *δ*=9.55*%*) and the error accounted for (Sign Test: *N*=1200, *Z*=−34.612, *p*< 0.001, weightings > timings, *δ*=20.1*%*) compared to the paired timing (Fig. 7).

**Fig. 8:**
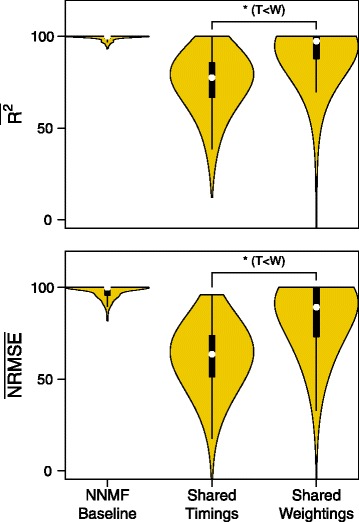
Reconstruction quality of timing and weightings shared across conditions. Variance accounted for and Error accounted for the fixed data set, where either the timing or the matrix data was assumed constant for all trials in both conditions. A Sign test showed that the fixed weightings had significantly higher reconstruction values than the timings. Although assuming fixed weighting lead to high variability in variance accounted for and error accounted for, the majority of the data could be reconstructed by assuming fixed weighting indicating a strong preference for recruiting similar muscle patterns during adaptation

Finally, the shared weighting set tested whether a single timing or weighting set could explain the variance of a data set consisting of all of the unpowered and powered condition data collected on that session. The shared weighting set had a statistically significant increase in the median variance accounted for (Sign Test: *N*=2400, *Z*=−34.497, *p*< 0.001, weightings > timings, *δ*=27.7*%*) and the error accounted for (Sign Test: *N*=2400, *Z*=−30.006, *p*< 0.001, weightings > timings, *δ*=33.2*%*) compared to the shared timing set (Fig. 8).

## Discussion

### The number of modules and their structure was preserved across exoskeleton assistance levels and training time

The results confirmed our hypothesis that the organization of the motor modules would not change between conditions. Six modules could explain the majority of the variance in EMG data during walking in a powered, bilateral ankle exoskeleton in both the unpowered and powered condition. Although 4 and 5 modules could explain a large portion of the data, Fig. 2 shows that 4 modules had a long tail, indicating that a substantial number of muscles had low *R*^2^ values. Our results are similar to previous research which showed that a set of 4 to 6 modules [13, 14, 20] could explain the majority of the variance in EMG data during unassisted walking. Although there were noticeable inter-subject differences in modules as shown in Fig. 1, the number of modules necessary to meet the median threshold of 90% was consistent across subjects.

The number of motor modules needed for an accurate reconstruction was preserved across conditions and the timings and weightings across conditions were more similar than random chance suggesting structural robustness of the modules. The similarities between conditions are striking given the biomechanical changes of the subjects highlighted in our previous work [35]. Although the high-level task (i.e. walking on a treadmill at a fixed speed) was not changed, the low-level strategy for achieving the task (i.e. the kinematics and kinetics of gait) and the energetic cost of walking did change substantially. On average, the subjects walked with greater plantarflexion, generated more positive work during plantarflexion, and lowered their energy expenditure in the powered condition.

The number of modules needed to reconstruct the data was also preserved with regard to training time in the exoskeleton. Previous studies on the effect of training and expertise on the organization of motor modules have conflicting results. One hypothesis is that experts, as a result of training, have more individual muscle control and less co-activation which requires a greater number of modules to capture. For example, in a balance beam task, an expert group of beam walkers showed more individual muscle control which required a greater number of modules than a novice group [60]. In contrast, expert rowers did not have any differences in module organization or muscle organization within modules when compared to novice rowers [61]. One interpretation is that basic movements (e.g walking) require less cortical involvement than highly trained tasks (e.g. balance beam walking) and do not show changes in module organization.

For a given number of modules (k = 6), we were able to explain greater variance in the EMG data during the powered condition compared to the unpowered condition, suggesting a reduction in the complexity of control during exoskeleton assistance. One potential explanation is that the amount of time in each condition was mismatched. Subjects walked for only 10 min in the unpowered condition before walking for 30 min in the powered condition. Because we compared the last 5 min of each trial, it is possible that the slight increase in reconstruction quality is due the greater amount of time adapting to exoskeleton assistance.

Although the number of subjects was sufficient for statistical significance of our hypotheses, this results of the study may be limited by the small sample size of 8. Furthermore, our testing protocol always had a short unpowered acclimation period followed by a longer powered period [37] and we did not randomize the order of conditions.

### The reconstruction quality of the extracted modules increased with training time

Although the organization of the motor modules did not change over the course of the training period, the reconstruction quality increased with training time. Using a linear mixed model regression for *R*^2^ and a module size of six, we found significant fixed effects for the training session and the trial number. This suggests that *R*^2^ may reflect improvement in the consistency of muscle recruitment due to both motor adaptation and motor consolidation [60, 62].

In the mixed-effects model, the estimated coefficient for training session was substantially larger than the coefficient for trial number demonstrating that *R*^2^ captured the importance of motor consolidation between training sessions relative to motor adaptation during a training session. Together, these results suggest that reconstruction quality could be a potential method for measuring adaptation during training in an exoskeleton.

### The timing and weighting data was similar between exoskeleton assistance levels

Following our hypothesis that the organization of motor modules would not change between conditions, we also expected that the timing and weighting data would be similar across conditions. In healthy subjects, previous research has shown that the timings of the motor modules are similar when subjects undergo whole-body kinetic changes (e.g. walking speed, bodyweight support [20], and incline [22]). Our results showed that motor modules are also similar when subjects undergo kinetic changes at individual joints. In contrast to those studies which only demonstrated similar timings across conditions, subjects walking in an exoskeleton showed substantial similarity in both timing and weighting data. Although the timing and weighting matrices across conditions were more similar than random chance (Fig. 3 and 5), our results showed that changes occurred in both the timing and weighting components (Figs. 6, 7, and 8). These results support the characterization of healthy walking as a robust task that can handle variations in parameters without large changes to the underlying coordination.

### Random weightings had better reconstruction than random timings

Similar to previous studies, we found that randomly generated data was able to reconstruct some of the EMG data recorded during walking [28]. The reconstruction quality measured via *r*^2^ and normalized root mean square error (NRMSE) was greater when the weighting matrices were randomized than when the timing matrices were randomized (Fig. 4). The set of randomly generated weighting data and optimized timing data produced a useful estimate (*R*^2^>0) but the randomly generated timing data and optimized weightings did not (*R*^2^<0). These results support the concept that gait variability is reflected more in the timing data of extracted modules than the weighting data [21].

### The weightings had higher similarity than the timings

For both within and between condition comparisons, the similarity between the extracted timing and weighting data was greater than that of random chance (Fig. 5). For the selected threshold of 75%, most of the timing data and almost all of the weighting data was similar. We found that, both within and between conditions, the weighting data had higher similarity than the timing data. This suggests that both natural variability and the variability due to perturbations by the exoskeleton were dominated by changes in the timing of recruitment not weightings.

### The weightings had higher reconstruction quality than the timings for the swapped, paired, and shared sets.

Although subjects reduced their energetic cost of walking and made substantial changes to the organization and effort of their joints during walking [35], the number, function, and organization of modules was preserved. Our results showed that assuming partial information from one condition could be used to reconstruct data from other conditions (Figs. 6, 7, and 8). Using a single set of muscle weightings (shared set), generalized across all conditions and training time, could account for the majority of the variance and error of all the measured data. Consistent with the idea of spinal circuity modulating the timing of activation, our results suggest that the muscle weighting data could be a low level construct which is shared and adapted by subjects when adapting to walking in the exoskeleton. These results agree with simulation studies that show that several different human movements pattern can potentially be synthesized from a small library of activation patterns [51].

One interpretation of these results is that the motor control strategy for walking in an exoskeleton is not based on high level changes to module organization but rather the strategy is based on making adjustments to the timing and weighting of individual muscles. This interpretation challenges one of major concepts in the hypothesis of motor modules, which is that they allow for reduction of the dimensionality of the control space [9, 63]. Our results agree with the hypothesis that there is a strong underlying pattern of coordination. When walking in an exoskeleton, the number of modules and the function of each model was preserved across exoskeletonassistance levels.

Following the hypothesis of motor modules, one would expect a scaling of muscles inside the module based on the original weighting ratio when the exoskeleton assistance level changes but muscles were adapted individually. Our study shows that subjects adapted to walking by changing both the module timing patterns and the module weightings. Our results agree with Steele et al. [32] that subjects are able to modulate both the timings and weightings of these modules in response to the assistance provided by the exoskeleton. The smaller number of modules they found for their study is likely the result of them using a smaller set of muscles (up to 8) and because their set included medial and lateral measurements of the soleus and gastrocnemius muscles. Differences in motor modules resulting from differences in exoskeleton design and control algorithm are a potential avenue of further study in order to help design future devices.

One potential reason for the similarity in coordination strategies is that the motor modules reflect the biomechanical constraints of the task. Previous research has shown that a similar reduction of motor signals to a lower-order dimensional space can be done on signals produced solely through task constraints and optimization of muscle activation signals [55, 56]. As our two conditions are both walking at a fixed speed, it is possible that the similarities in the motor modules are driven by biomechanical task constraints. If the subject’s preferred gait in an exoskeleton were to causes greater changes to the biomechanics of the task, it is possible that greater changes in muscle recruitment and coordination would be necessary.

Another potential reason for the motor module similarity is that the central nervous system manages control complexity by searching in the area around a previously found solution. The similarity in the subject’s preferred solution to the new walking task may reflect recall of a “good-enough” robust solution learned over years of walking rather than a online generated “optimal” solution [64, 65]. Motor modules may also reflect neuromechanical solutions that have been chosen for their generalization rather than specificity [60, 66]. An “optimal” solution, that is singular, may not be ideal because it lacks the robustness that can be conferred by generalizing and selecting from modules that can be used for multiple biomechanical tasks [67]. Similarly to the preservation of modules during adaptation to an isometric task in the upper arm [26, 27], the subject’s adaptations to the exoskeleton may be most compatible with their current knowledge and easier to learn by being similar to the original modules.

Understanding what portions of the muscle recruitment patterns are preserved during walking and what portions reflect responses to dynamic perturbations could help develop algorithms for assistive devices to respond fluidly and rapidly to the user in real-world walking environments. We speculate that there may be an advantage to analyzing human movement in modules that extends beyond the idea of simple reduction. It is possible that preservation of motor modules measured during walking in an ankle exoskeleton could also be found for devices spanning other joints leading to greater understanding of whole body coordination in an exoskeleton.

## Conclusion

We investigated muscle recruitment patterns during walking in a powered bilateral ankle exoskeleton and found the organization (module size and function) did not change between powered and unpowered conditions. Furthermore, across conditions, the timings and weightings were more similar than would be expected by random chance suggesting that module components were preserved across assistance levels. We found that for a fixed module size, the reconstruction quality of NNMF of the subject’s muscle signal data improved over time, suggesting that training resulted in greater consistency of muscle recruitment. The weighting data could better reconstruct EMG signals across conditions than the timing data suggesting that adaptation to walking in an exoskeleton was dominated by changes in timing of the modules.

## Abbreviations

BF: Biceps femoris long head
EMG: Electromyography
GX: Gluteus maximus
MG: Medial gastrocnemius
NNMF: Non-negative matrix factorization
NRMSE: Normalized root mean square error
PER: Peroneus longus
RF: Rectus femoris
SM: Semitendenosis
SOL: Soleus
TA: Tibialis anterior
VAF: Variance accounted for
VL: Vastus lateralis
VM: Vastus medialis
